# LACTATE IMPAIRS VASCULAR PERMEABILITY BY INHIBITING HSPA12B EXPRESSION *VIA* GPR81-DEPENDENT SIGNALING IN SEPSIS

**DOI:** 10.1097/SHK.0000000000001983

**Published:** 2022-09-30

**Authors:** Min Fan, Kun Yang, Xiaohui Wang, Xia Zhang, Jingjing Xu, Fei Tu, P. Spencer Gill, Tuanzhu Ha, David L. Williams, Chuanfu Li

**Affiliations:** ∗ Department of Surgery, James H. Quillen College of Medicine, East Tennessee State University, Johnson City, Tennessee; † Center of Excellence in Inflammation, Infectious Disease and Immunity, East Tennessee State University, Johnson City, Tennessee

**Keywords:** Heat shock protein A12B, lactate, polymicrobial sepsis, vascular permeability, VE-cadherin

## Abstract

**Introduction**: Sepsis impaired vascular integrity results in multiple organ failure. Circulating lactate level is positively correlated with sepsis-induced mortality. We investigated whether lactate plays a role in causing endothelial barrier dysfunction in sepsis. **Methods**: Polymicrobial sepsis was induced in mice by cecal ligation and puncture (CLP). Lactic acid was injected i.p. (pH 6.8, 0.5 g/kg body weight) 6 h after CLP or sham surgery. To elucidate the role of heat shock protein A12B (HSPA12B), wild-type, HSPA12B-transgenic, and endothelial HSPA12B-deficient mice were subjected to CLP or sham surgery. To suppress lactate signaling, 3OBA (120 μM) was injected i.p. 3 h before surgery. Vascular permeability was evaluated with the Evans blue dye penetration assay. **Results**: We found that administration of lactate elevated CLP-induced vascular permeability. Vascular endothelial cadherin (VE-cadherin), claudin 5, and zonula occluden 1 (ZO-1) play a crucial role in the maintenance of endothelial cell junction and vascular integrity. Lactate administration significantly decreased VE-cadherin, claudin 5, and ZO-1 expression in the heart of septic mice. Our *in vitro* data showed that lactate (10 mM) treatment disrupted VE-cadherin, claudin 5, and ZO-1 in endothelial cells. Mechanistically, we observed that lactate promoted VE-cadherin endocytosis by reducing the expression of HSPA12B. Overexpression of HSPA12B prevented lactate-induced VE-cadherin disorganization. G protein–coupled receptor 81 (GPR81) is a specific receptor for lactate. Inhibition of GPR81 with its antagonist 3OBA attenuated vascular permeability and reversed HSPA12B expression in septic mice. **Conclusions**: The present study demonstrated a novel role of lactate in promoting vascular permeability by decreasing VE-cadherin junctions and tight junctions in endothelial cells. The deleterious effects of lactate in vascular hyperpermeability are mediated *via* HSPA12B- and GPR81-dependent signaling.

## INTRODUCTION

Sepsis is defined as a dysregulated inflammatory response induced by an infection, which is associated with life-threating organ dysfunction (1,2). Endothelial hyperpermeability in response to sepsis and septic shock is a major contributor to multiple organ failure (3,4). Adherens junctions and tight junctions play an important role in regulating endothelial barrier integrity (5). Adhesion of vascular endothelial cadherin (VE-cadherin), a key component of adherens junctions, is the major adhesion process in vascular development (5,6). Insufficient expression of the tight junction molecules, claudin 5 and zonula occluden 1 (ZO-1), disrupts endothelial cell-cell connection and increases endothelial permeability (7,8).

As a biomarker of septic shock, clinical studies reveal that serum lactate levels are closely associated with the morbidity and mortality of sepsis (9). Inhibition of aerobic glycolysis by either PKM2 (pyruvate kinase M2) (10) or 2-deoxy-d-glycose (11) decreases lactate production and improves survival rate in experimental model of sepsis induced by cecal ligation and puncture (CLP). In addition, our recent study has revealed that lactate promotes the production of inflammatory cytokine TNF-α and IL-6 (12), as well as release of exosomal HMGB1 (high-mobility group box 1) from macrophage, which can induce vascular endothelium permeability in experimental sepsis (13). However, whether lactate has a direct effect on endothelial permeability remains unclear.

Heat shock protein A12B (HSPA12B), a member of HSP70 family, is predominantly expressed in endothelium and required for angiogenesis (14,15). Our group has uncovered that deficiency of endothelial HSPA12B impairs angiogenesis in a mouse model of myocardial infarction (16) and induces intercellular adhesion molecule 1 and vascular cell adhesion molecule 1 expression in experimental sepsis (17). On the contrary, overexpression of HSPA12B promotes endothelial cell angiogenesis and attenuates adhesion molecular expression. Little is known about the role of HSPA12B in endothelial adherens junction and tight junction and its relationship with lactate in polymicrobial sepsis.

In the current study, we investigated whether lactate plays a role in endothelial permeability after sepsis and, if so, by what mechanism(s). We found that administration of lactate or depletion of endothelial cell HSPA12B exacerbates sepsis-induced organ dysfunction and vascular hyperpermeability by impairing VE-cadherin junctions and tight junctions. Importantly, we observed that lactate reduced HSPA12B expression, and HSPA12B is required for lactate-regulated VE-cadherin junctions and tight junctions in endothelial cells. In addition, inhibition of lactate signaling reverses HSPA12B expression and improves vascular integrity after sepsis.

## MATERIALS AND METHODS

### Animals

Endothelial cell–specific HSPA12B-deficient (eHSPA12B^−/−^) mice and HSPA12B-transgenic (HSPA12B-Tg) mice were constructed as described in our previous studies (16,18). Wild-type (WT) C57BL/6 mice were purchased from Jackson Laboratory (Indianapolis, IN). All mice were maintained and bred at the Division of Laboratory Animal Resources at East Tennessee State University (ETSU). All experimental procedures were performed in accordance with the Guide for the Care and Use of Laboratory Animals published by the National Institutes of Health (NIH Publication, eighth edition, 2011) and approved by the ETSU Committee on Animal Care (A3203-01).

### Polymicrobial sepsis model

Male mice (8–10 weeks) were subjected to CLP to induce polymicrobial sepsis as previously described (13). Briefly, mice were anesthetized and maintained by inhalation of 1.5% to 2% isoflurane driven by 100% oxygen flow. Following midline incision, the mouse cecum was ligated between the third and fourth vascular arcade with a 4-0 silk suture and punctured with a 25-gauge needle. Sham operation is performed by isolation of the cecum without ligation and puncture. A single dose of resuscitative fluid saline was administered by s.c. injection after surgery. Mice were recovered in prewarmed cages (37°C).

### *In vivo* treatments

To investigate whether elevated serum lactate levels contribute to vascular barrier dysfunction, lactic acid (Millipore Sigma, St. Louis, MO; pH 6.8, 0.5 g/kg body weight) was injected i.p. 6 h after surgery. The dose of lactate is adjusted based on a previous publication (19) and was tested on our recent publications (12,13). In addition, this dose of lactate is nonlethal and does not cause acidosis (20). Serum lactate levels were measured by Lactate Assay Kit (Millipore Sigma; MAK064). In separate experiments, 3-hydroxybutyrate (3OBA) (Millipore Sigma; 120 μM) was administered to mice 3 h before CLP or sham surgical operation to block G protein–coupled receptor 81 (GPR81) signaling. The dose of 3OBA was determined based on our pilot study and previous report (13).

### Echocardiography

We performed echocardiography on mice 24 h after CLP to test cardiac function according to our previous studies (18,21). Left ventricular (LV) wall thickness, LV end-systolic diameter, and LV end-diastolic diameter were examined by M-mode tracings. Thereafter, percent fractional shortening (FS%) and ejection fraction (EF%) were calculated.

### Organ injury measurement

Serum creatinine (Millipore Sigma; MAK080) and aspartate aminotransferase (AST) (Millipore Sigma; MAK055) levels were measured by commercial kits according to the manufacturer's protocols.

### Vascular permeability assay

*In vivo* vascular permeability was assessed by the Evans blue dye (EBD) assay. Twenty-four hours after CLP or sham surgery, 0.5% EBD (in phosphate-buffered saline [PBS]) was injected *via* penile vein 30 min before mice were killed. Subsequently, mice were perfused with PBS *via* left ventricle to remove the intravascular dye. Livers and kidneys were removed and dried overnight. Evans blue dye was extracted in formamide by incubation of tissues overnight at 55°C. Evans blue dye levels in tissue supernatant were measured by spectrophotometric analysis at 610 nm (22,23).

### Endothelial cell culture

Human umbilical vein endothelial cells (HUVECs) (ATCC, PCS-100-013) were cultured in vascular cell basal medium (ATCC, Manassas, VA; PCS-100-030) supplemented with growth factors and 5% fetal bovine serum at 37°C with 5% CO_2_. Human umbilical vein endothelial cells were treated with 10 mM l-lactic acid for 2 or 6 h.

### Cell transfection

Human umbilical vein endothelial cells were cultured to 70% confluence and were transfected with adenoviral HSPA12B (Ad-HSPA12B, MOI = 10) or adenoviral GFP (Ad-GFP). 6 h after transfection, cells were washed with PBS and incubated with fresh medium overnight before lactate treatment.

### Immunofluorescent staining

Heart tissues were collected after the mice were killed, fixed in 4% paraformaldehyde, embedded in tissue processing medium (O.C.T.), and cut horizontally at a 10-mm thickness. The slides were stained with specific anti–VE-cadherin antibody (1:200 dilution, sc-9,989; Santa Cruz Biotechnology, Santa Cruz, CA) at 4°C for overnight. Human umbilical vein endothelial cells obtained from *in vitro* cell culture were fixed with 3.7% formaldehyde for 20 min and permeabilized with 0.1% Triton X-100 for 20 min, followed by blocking with 3% bovine serum albumin for 30 min at room temperature. Subsequently, cells were incubated with anti–VE-cadherin antibody (1:200 dilution, sc-9,989; Santa Cruz Biotechnology) at 4°C for overnight. Alexa Fluor 594 secondary antibody (1:500 dilution in PBS, A-11005; Invitrogen) was added to the section to visualize the staining. DAPI (blue) was used to counterstain the nuclei. The stained sections and cells were measured using Confocal Microscope (Leica Camera, Wetzlar, Germany).

### Enzyme-linked immunosorbent assay

The serum levels of proinflammatory cytokines (TNF-α and IL-6) following sepsis were measured using commercially available ELISA kits (PeproTech, Cranbury, NJ) according to the instruction provided by the manufacturer.

### Western blot

Western blot was performed as described previously (7). Briefly, cellular proteins were prepared in ice-cold RIPA buffer containing protease inhibitor and quantified with Pierce BCA protein assay kit (23,225; ThermoFisher, Waltham, MA). Proteins were separated on 10% sodium dodecyl sulfate–polyacrylamide gel electrophoresis and transferred onto nitrocellulose blotting membranes (10,600,003; GE Healthcare, Chicago, IL). The membranes were incubated with appropriate primary antibodies (1:1,000 dilution in 5% bovine serum albumin) followed by the peroxidase-conjugated secondary antibody (1:5,000 dilution in 5% nonfat milk). The signals were quantified using G:Box gel imaging system by Syngene (Bangalore, India). The following primary antibodies were used in the present study: anti–VE-cadherin antibody (1:200 dilution, sc-9,989; Santa Cruz Biotechnology), anti-HSPA12B antibody (1:1,000 dilution, a gift from Dr. Zhihua Han [ETSU, Johnson City, TN]), anti-claudin 5 antibody (1:1,000 dilution, ab131259; Abcam, Cambridge, United Kingdom), anti–ZO-1 antibody (1:1,000 dilution, 13,663 s; Cell Signaling Technology, Danvers, MA), anti–β-actin antibody (1:1,000 dilution; 3,700 s; Cell Signaling Technology), and anti-GAPDH antibody (1:1,000 dilution, Cell Signaling Technology, 2,118 s).

### Quantitative real-time polymerase chain reaction

Total mRNA was isolated from cultured cells using RNAzol RT (RN 190; Molecular Research Center, Cincinnati, Ohio) in accordance with the manufacturer's protocol as described previously (24). The mRNA was converted to cDNA by High Capacity cDNA Transcription kit (4,368,814; Applied Biosystems, Waltham, MA). The mRNA levels of VE-cadherin, claudin 5, and ZO-1 were measured using SYBR green mix (KCQS00; Sigma-Aldrich, St. Louis, MO) system and quantified with the two (−ΔΔct) relative quantification methods that were normalized to β-actin.

### Statistical analysis

Data are expressed as mean ± SD. Comparisons of data between groups were made using two-tailed *t* test or one-way ANOVA followed by Tukey procedure for multiple range tests. *P* < 0.05 was considered to be significant.

## RESULTS

### Lactate induces organ dysfunction and vascular permeability in sepsis

**Fig. 1:**
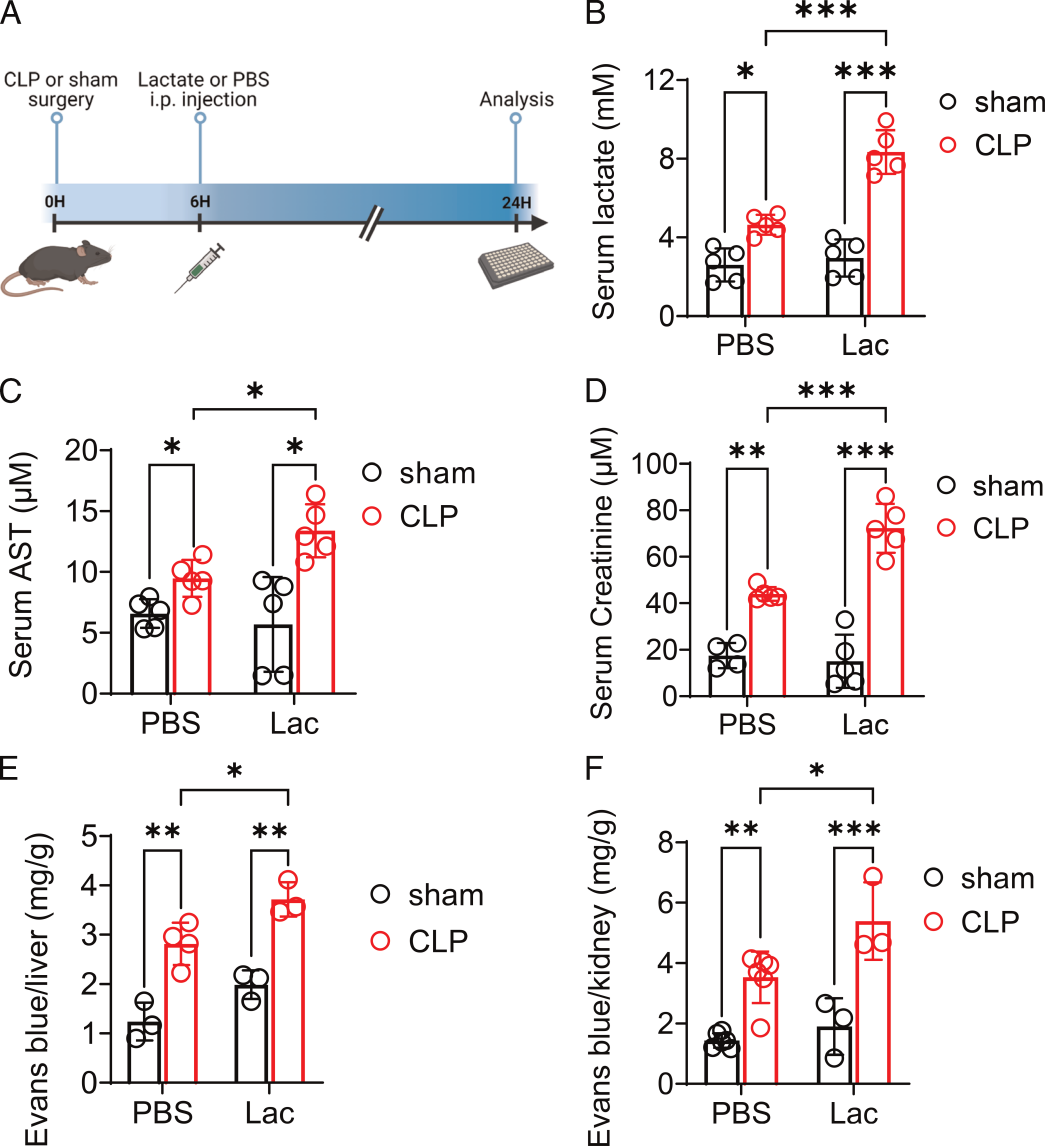
**Lactate administration induced organ dysfunction and vascular permeability after CLP sepsis.** Mice were subjected to CLP or sham surgery followed by PBS or lactic acid (pH 6.8, 0.5 g/kg body weight) i.p. injection. Twenty-four hours later, blood was collected (A), and serum was isolated to examine serum lactate (B), AST (C), and creatinine (D) levels. E–F, In separate experiments, 0.5% Evans blue was injected *via* penile vein 30 min before mice were killed. Vascular permeability of liver and kidney was measured. n = 3–5/group. *P < 0.05, **P < 0.01, ***P < 0.001 compared with indicated group.

Accumulating evidence has revealed that increased lactate levels correlate with multiple organ dysfunction in septic patients (9,25,26). To investigate whether lactate is involved in the development of organ dysfunction during sepsis, we administered lactate (0.5 g/kg body weight) *via* i.p. injection 6 h after CLP or sham surgery, and tissues were collected for subsequent analysis (Fig. 1A). We observed that CLP strongly induced neutrophil infiltration in the spleen (Fig. S1A, http://links.lww.com/SHK/B501) and increased serum levels of TNF-α and IL-6 (Fig. S1, B and C, http://links.lww.com/SHK/B501) as compared with sham mice. In consistent with previous reports, CLP increased serum lactate levels (Fig. 1B) and induced liver and kidney injury as evidenced by increased serum AST (6.58 ± 1.17 vs. 9.47 ± 1.51) and creatinine (17.5 ± 5.43 vs. 44.0 ± 2.95) levels when compared with sham group (Fig. 1, C and D) (11,27). Importantly, lactate supplementation further increased serum AST and creatinine levels by 41.4% and 64.3%, respectively, when compared with CLP mice with PBS supplementation (Fig. 1, C and D). In addition, lactate administration suppressed proinflammatory cytokine (TNF-α and IL-6) production following sepsis (Fig. S1, B and C, http://links.lww.com/SHK/B501), which is consistent with our previous findings (12). These data suggest that lactate promotes organ dysfunction during sepsis. Vascular leakage leads to tissue edema and impaired vascular perfusion, which consequently contributes to organ dysfunction in critical illness, such as sepsis (3,28). Next, we assessed whether lactate affected vascular permeability after CLP sepsis by EBD assay (22). As shown in Figure 1, E and F, CLP increased vascular permeability in liver and kidney as evidenced by increased EBD penetration to the tissues. Supplementation of lactate further increased liver and kidney vascular permeability in sepsis mice (Fig. 1, E and F). Notably, lactate supplementation did not significantly increased serum lactate levels in sham-operated mice (Fig. 1B), which is in agreement with a recent study showing that lactate levels were back to baseline concentrations 30 min after lactate i.p. injection (19). Clearance of lactate from the circulation is primarily mediated by the liver (60%) and kidney (30%) (29). Lactate is metabolized and cleared rapidly in sham-operated mice with liver and kidney function (Fig. 1, C and D). Therefore, lactate supplementation did not cause vascular permeability in sham mice (Fig. 1, E and F). Together, our data suggest that lactate induces vascular permeability and contributes to organ dysfunction in sepsis.

### Lactate impairs VE-cadherin junctions and tight junctions in endothelial cells

**Fig. 2:**
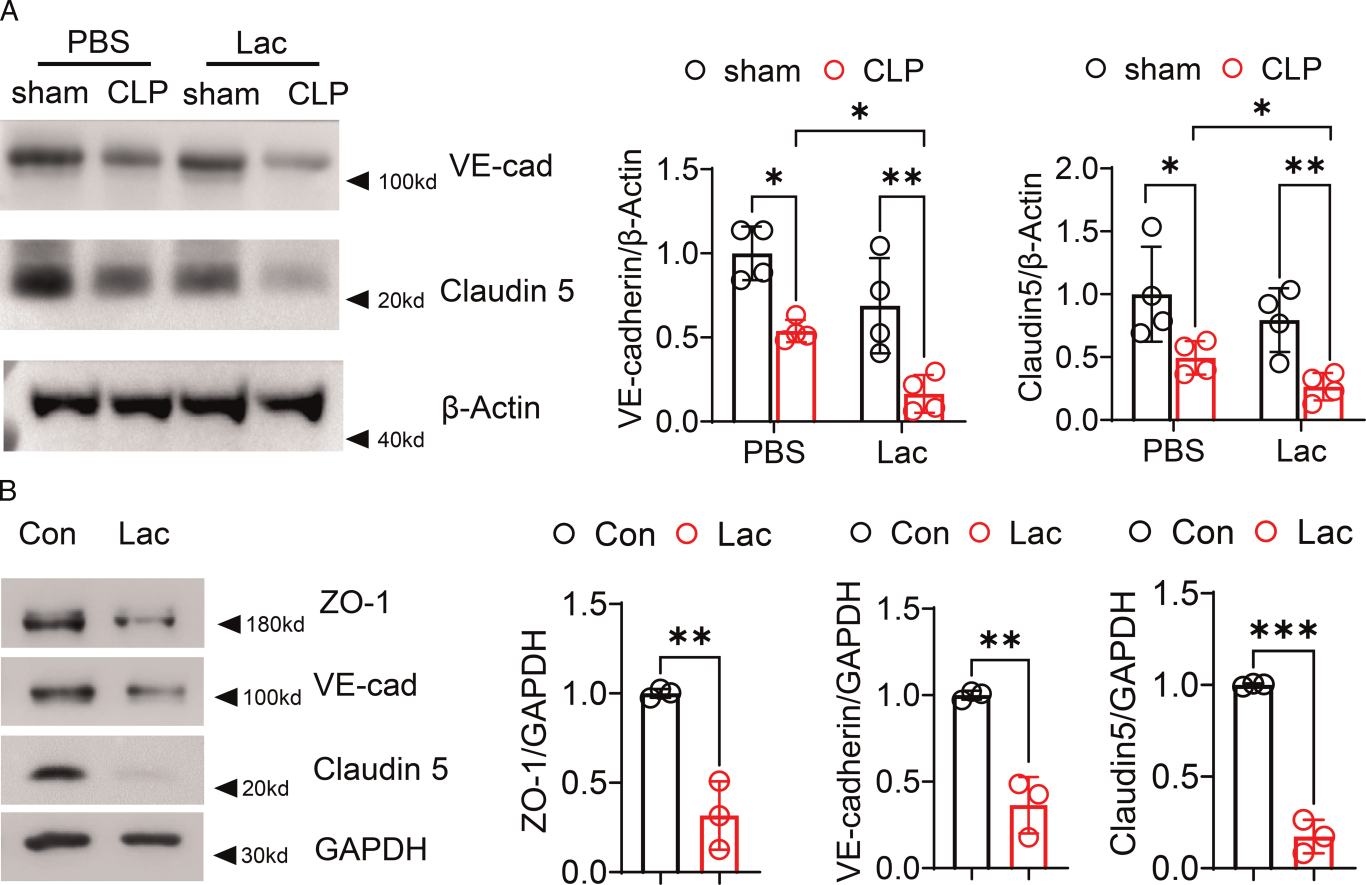
**Lactate administration decreased VE-cadherin junctions and tight junctions after CLP**. A, Mice were subjected to CLP or sham surgery followed by PBS or lactic acid (pH 6.8, 0.5 g/kg body weight) i.p. injection. Twenty-four hours later, heart tissues were collected and protein was isolated. The levels of VE-cadherin and claudin 5 in the myocardium were examined by Western blot. n = 4/group. B, Human umbilical vein endothelial cells were treated with l-lactic acid (10 mM) for 6 h, and protein of HUVECs was then isolated. The expression of VE-cadherin, claudin 5, and ZO-1 was examined by Western blot. n = 3/group. *P < 0.05, **P < 0.01, ***P < 0.001 compared with indicated group.

As a major adherens junctional protein, VE-cadherin plays a crucial role in maintaining endothelial cell connection and stability (30,31). We next determined whether lactate can alter VE-cadherin integrity. As shown in Figure 2A, there was a reduction of VE-cadherin expression in septic hearts, when compared with the sham control group. Moreover, lactate treatment further disrupted the expression of VE-cadherin after sepsis (Fig. 2A). Besides adherens junctions, tight junctions are also important in maintaining organ integrity (32). We examined the expression of the tight junction protein claudin 5 in the heart and detected lower claudin 5 expression with lactate treatment than septic control mice (Fig. 2A), indicating that lactate exerts an additive effect by impairing tight junctions in the myocardium after sepsis.

To further confirm the role of lactate in endothelial cell function, we treated HUVECs with lactate *in vitro* and investigated the expression of VE-cadherin, claudin 5, and ZO-1. In accordance with our *in vivo* finding, lactate administration decreased VE-cadherin, claudin 5, and ZO-1 levels in endothelial cells (Fig. 2B).

### Lactate suppresses HSPA12B expression in endothelial cells

**Fig. 3:**
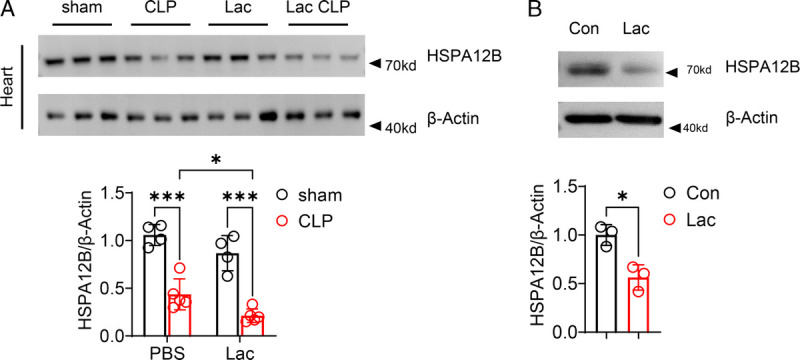
**Administration of lactate eliminated HSPA12B expression following sepsis**. A, Mice were subjected to CLP or sham surgery followed by PBS or lactic acid (pH 6.8, 0.5 g/kg body weight) i.p. injection. Twenty-four hours later, heart tissues were collected, and protein was isolated. The levels of HSPA12B in the myocardium were examined by Western blot. n = 4/group. B, Human umbilical vein endothelial cells were treated with l-lactic acid (10 mM) for 6 h, and protein of HUVECs was then isolated. The expression of HSPA12B was examined by Western blot. n = 3/group. *P < 0.05, ***P < 0.001 compared with indicated group.

Heat shock protein A12B is a recently discovered protein that is primarily expressed in endothelial cells (14,15). We and others have previously shown that HSPA12B is required for endothelial cell angiogenesis and adhesion (15,16,33). We found that the expression of HSPA12B in the heart decreased remarkably after CLP sepsis (Fig. 3A). Interestingly, treatment with supplemental lactate further downregulated HSPA12B expression following sepsis (Fig. 3A). In addition, administration of lactate disrupted HSPA12B expression in endothelial cells (Fig. 3B). Taken together, our findings suggest that HSPA12B may play a critical role in lactate-impaired endothelial barrier leakage.

### Endothelial cell–specific deletion of HSPA12B exacerbates organ dysfunction and promotes vascular hyperpermeability after sepsis

**Fig. 4:**
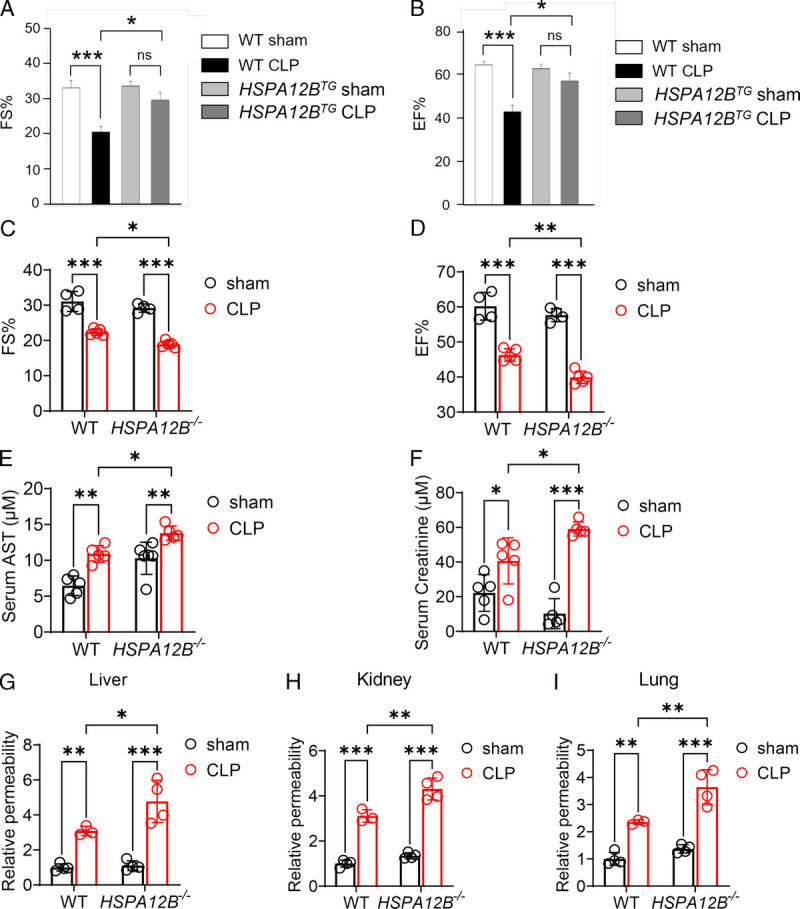
**Endothelial cell–specific depletion of HSPA12B elevated CLP-induced organ dysfunction and endothelial hyperpermeability**. Wild-type, HSPA12B-Tg and endothelial HSPA12B knockout (eHSPA12B^−/−^) mice were subjected to sham or CLP surgical operation. A–D, Cardiac function was examined by echocardiography 24 h after surgery. E and F, Serum AST and creatinine levels were examined 24 h after surgery. G–I, In separate experiments, 0.5% Evans blue was injected *via* penile vein 30 min before mice were killed. Vascular permeability of liver, kidney, and lung was measured. n = 4–5/group. *P < 0.05, **P < 0.01, ***P < 0.001 compared with indicated group.

Next, we induced CLP sepsis in WT, HSPA12B-Tg, and endothelial cell–specific eHSPA12B^−/−^ mice and tested cardiac function 24 h after surgery. Cecal ligation and puncture reduced FS% and EF% (Fig. 4, A–D). However, overexpression of HSPA12B reversed FS% and EF% levels after CLP (Fig. 4, A and B). In contrast, depletion of endothelial cell HSPA12B further suppressed FS% and EF% levels (Fig. 4, C and D). These data indicate that HSPA12B is required for maintenance of cardiac function after sepsis. Moreover, serum AST and creatinine levels were higher in eHSPA12B^−/−^ CLP mice than in WT CLP mice (Fig. 4, E and F), suggesting that inhibition of HSPA12B worsens liver and kidney injury after sepsis.

**Fig. 5:**
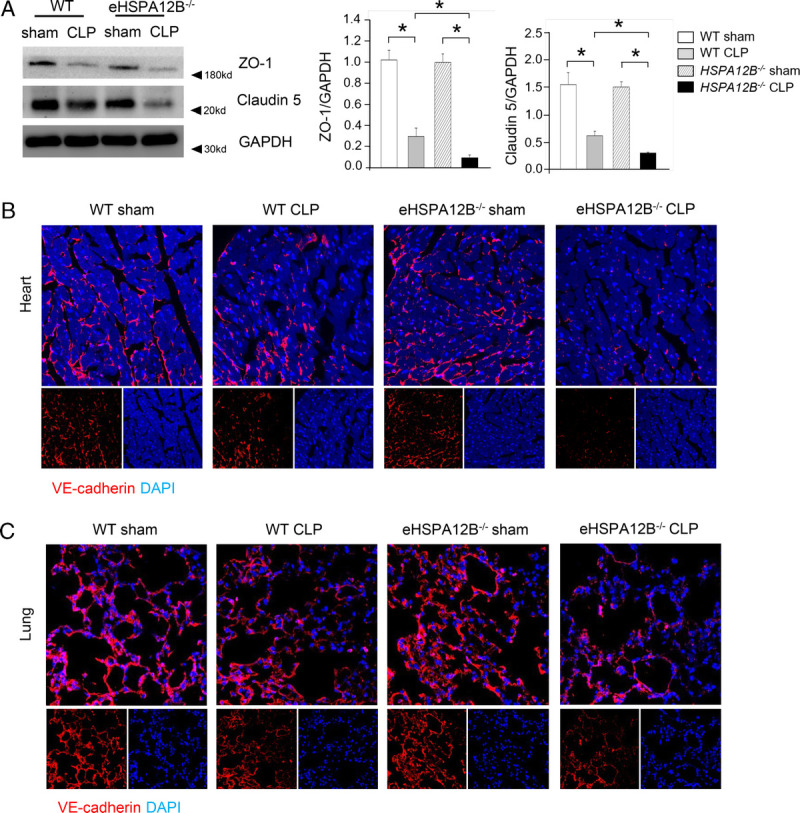
**HSPA12B is required for maintaining VE-cadherin junctions and tight junctions after sepsis**. Wild-type and eHSPA12B^−/−^ mice were subjected to sham or CLP surgery. Twenty-four hours later, heart and lung tissues were collected. A, The levels of claudin 5 and ZO-1 were examined by Western blot. Immunofluorescent staining was performed to investigate the integrity of VE-cadherin in the heart (B) and lung (C). n = 4–5/group. *P < 0.05 compared with indicated group.

We then addressed whether HSPA12B participates in vascular permeability following sepsis. Figure 4, G–I, shows that HSPA12B deficiency enhanced sepsis-induced endothelial hyperpermeability. In addition, the levels of claudin 5 and ZO-1 were significantly lower in eHSPA12B^−/−^ hearts than in the heats of WT septic mice (Fig. 5A). Figure 5, B and C, shows that the positive staining of VE-cadherin was decreased in both hearts and lung of eHSPA12B^−/−^ mice after CLP, when compared with WT CLP mice. These data reveal that HSPA12B is required for maintaining tight junctions and VE-cadherin junctions after sepsis.

### HSPA12B is required for lactate-decreased VE-cadherin junctions and tight junctions in endothelial cells

**Fig. 6:**
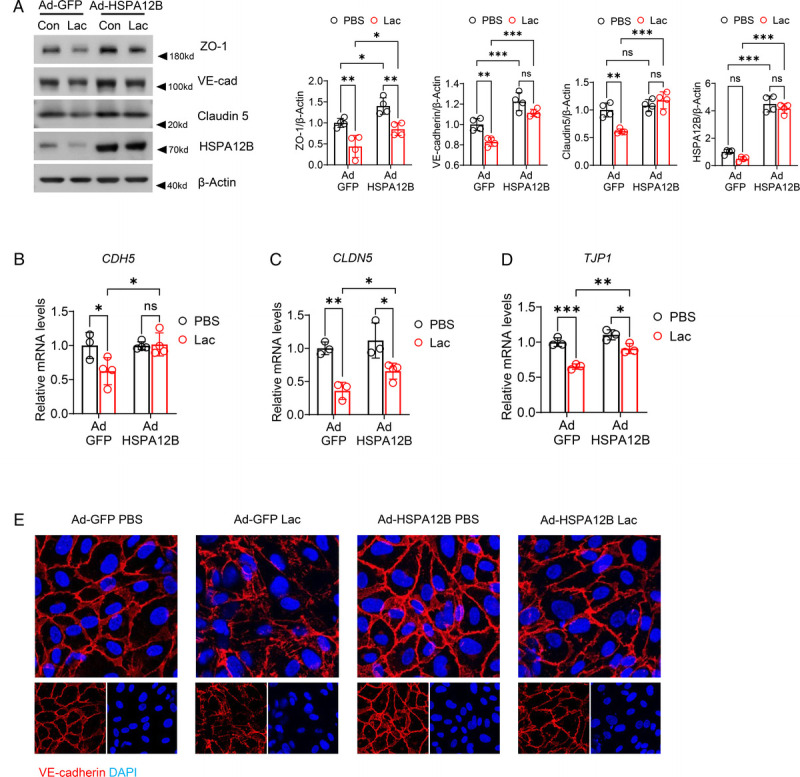
**HSPA12B is required for lactate-decreased VE-cadherin junctions and tight junctions in endothelial cells**. Endothelial cells were transfected with Ad-HSPA12B or Ad-GFP followed by lactate administration (10 mM). The expression of VE-cadherin, claudin 5, and ZO-1 was examined by Western blot (A) and quantitative real-time polymerase chain reaction (B–D). E, The integrity of VE-cadherin in endothelial cells was measured by immunofluorescent staining. n = 3–4/group. *P < 0.05, **P < 0.01, ***P < 0.001 compared with indicated group.

To elucidate whether HSPA12B plays a role in lactate-induced endothelial permeability, we transfected endothelial cells with adenoviral HSPA12B (Ad-HSPA12B) or Ad-GFP before lactate administration. As shown in Figure 6A, overexpression of HSPA12B attenuated the downregulation of VE-cadherin, claudin 5, and ZO-1 expression induced by lactate. Similarly, transfection of Ad-HSPA12B upregulated mRNA levels of VE-cadherin, claudin 5, and ZO-1 that were repressed by lactate (Fig. 6, B–D). When compared with Ad-GFP transfection, the integrity of VE-cadherin was reversed by Ad-HSPA12B transfection after lactate treatment (Fig. 6E). These results showed that HSPA12B is required for lactate-regulated VE-cadherin junctions and tight junctions.

### Inhibition of GPR81/lactate signaling induces HSPA12B expression decreased by sepsis and improves vascular integrity

**Fig. 7:**
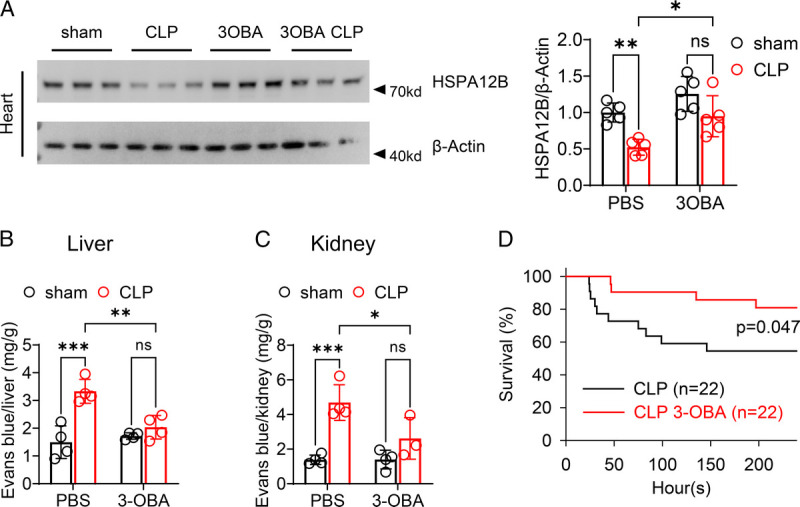
**Administration of 3OBA protected against CLP blunted HSPA12B expression, induced hyperpermeability and mouse death rate**. Mice were treated with PBS or 3OBA (120 μM) by i.p. injection 3 h before subjecting to CLP or sham surgery followed. A, Twenty-four hours later, heart tissues were collected, and protein was isolated. The levels of HSPA12B in the myocardium were examined by Western blot. n = 5/group. B and C, Evans blue 0.5% was injected *via* penile vein 30 min before mice were killed. Vascular permeability of liver and kidney were measured. n = 3–4/group. D, In separate experiments, mouse survival rate was measured (n = 22/group). *P < 0.05, **P < 0.01, ***P < 0.001 compared with indicated group.

Lactate is regarded as a signaling molecule that interacts with GPR81 (34). To determine the role of GPR81/lactate signaling, mice were i.p. injected of a GPR81 inhibitor, 3OBA (35), before CLP or sham surgery. Administration of 3OBA enhanced HSPA12B expression in the presence of sepsis (Fig. 7A). In addition, 3OBA treatment attenuated sepsis-induced liver and kidney EBD penetration (Fig. 7, B and C), suggesting that inhibition of GPR81/lactate signaling improves vascular permeability after sepsis. Moreover, 3OBA administration also improved survival outcomes of septic mice (Fig. 7D).

## DISCUSSION

In this study, we describe a role for lactate as a mediator of vascular permeability and multiple organ dysfunction in sepsis. Our findings show that lactate disrupts VE-cadherin integrity and decreases the expression of VE-cadherin and tight junction proteins (claudin 5 and ZO-1) in endothelial cells. We also observed that administration of supplemental lactate exacerbated sepsis-induced decreases in HSPA12B expression. Endothelial cell–specific deletion of HSPA12B exhibited vascular hyperpermeability *via* downregulation of VE-cadherin and tight junction protein levels and worsened multiple organ dysfunction during polymicrobial sepsis. In contrast, adenovirus-mediated overexpression of HSPA12B attenuated the effect of lactate on VE-cadherin, claudin 5, and ZO-1 in endothelial cells. Importantly, pharmacological inhibition of lactate/GPR81 signaling attenuated sepsis-induced HSPA12B downregulation and vascular permeability and improved survival outcome of septic mice. This suggests that it may be possible to prevent and/or treat lactate-induced organ injury and mortality in sepsis.

Sepsis is a complex syndrome initiated by infection and characterized by multiple organ dysfunction. Vascular endothelial damage, resulting from the invading microbes and systemic proinflammation, contributes to the pathogenesis of multiple organ dysfunction during sepsis (36–38). Therefore, understanding the mechanisms that lead to vascular injury would be an important advance in sepsis management.

We and others have demonstrated that glycolysis-derived lactate is a critical effector metabolite that triggers various cell signaling pathways in regulating innate immune responses during experimental sepsis (12,13,20,27,39). Of note, a recent study by Khatib-Massalha et al. (20) shows that elevated levels of bone marrow lactate increase bone marrow vascular permeability, leading to enhanced neutrophil mobilization during infection. To examine whether systemic elevation of lactate levels could induce vascular permeability, mice were subjected to CLP-induced sepsis followed by i.p. administration of lactate. We observed that CLP-induced vascular permeability in the liver and kidney tissues. Lactate supplementation further promoted vascular permeability in CLP sepsis mice. Consistent with these observations, our data show that lactate supplementation worsened CLP-induced liver and kidney dysfunction.

Previous studies have reported that HSPA12B is required to maintain endothelium homeostasis in the mouse model of sepsis and sepsis-induced cardiovascular diseases (17,18,40). In addition, it is reported that the circulating HSPA12B levels are higher in patients with severe sepsis (patients with at least one organ dysfunction within 24 h after inclusion, n = 66) than in patients with sepsis (n = 21), suggesting that HSPA12B could be a biomarker of endothelial cell injury in sepsis (41). In the present study, we found that HSAP12B is an essential mediator in lactate-induced downregulation of VE-cadherin, claudin 5, and ZO-1 in endothelial cells. Of greater significance, genetic depletion of HSPA12B in endothelial cells renders mice more susceptible to sepsis-induced downregulation of adhesion molecules and tight junction proteins, vascular hypermutability and multiple organ dysfunction. This observation is consistent with our recent studies showing that endothelial cell–specific deletion of HSPA12B increased mortality rate of septic mice (17,40). Conversely, adenovirus-mediated overexpression of HSPA12B preserved the expressions of VE-cadherin, claudin 5, and ZO-1 in lactate-treated endothelial cells. In addition, pharmacologically inhibiting the lactate receptor, GPR81, attenuated sepsis-induced decreases in HSPA12B expression, vascular permeability, and improved survival outcome of septic mice.

Several limitations are presented in our present study. Although we show that overexpression of HSPA12B preserved the expression of VE-cadherin, claudin 5, and ZO-1 in lactate-treated endothelial cells, the underlying mechanisms are unclear. Our previous study revealed that HSPA12B cooperates with transcription regulator YAP in regulating the transcription of genes associated with angiogenesis (16). It is reported that YAP regulates adhesion junction dynamics in endothelial cells and is required for the formation of barrier integrity of endothelium (42,43). Therefore, it is intriguing to speculate the involvement of YAP signaling in lactate-induced vascular permeability in future studies. In addition, accumulating evidence indicates that lactate regulates the response of immune cells to infection (12,13,44). Therefore, it is imperative to investigate whether lactate could alter immune cell functions, such as bacterial clearance, thereby consequently contributing to vascular permeability.

In conclusion, the findings of this study delineate the essential role of lactate in promoting vascular permeability by downregulating HSPA12B in endothelial cells during sepsis. Suppression of lactate/GPR81 signaling is effective in reducing vascular permeability and improving organ function in polymicrobial sepsis. Therefore, our study not only provides molecular basis for the lactate as critical biomarker in sepsis prognosis but also suggests that blocking lactate/GPR81 signaling by GPR81 inhibitors may prevent lethality in sepsis.

## Supplementary Material

SUPPLEMENTARY MATERIAL
