# Supplementary material for: LACTATE IMPAIRS VASCULAR PERMEABILITY BY INHIBITING HSPA12B EXPRESSION VIA GPR81-DEPENDENT SIGNALING IN SEPSIS
Source: Shock. 2022 Sep 30;58(4):304–12. doi: 10.1097/SHK.0000000000001983 (PMC9584042; doi:10.1097/SHK.0000000000001983)
Supplement: SUPPLEMENTARY MATERIAL [file shock-58-304-s002.docx]

**LACTATE IMPAIRS VASCULAR PERMEABILITY BY INHIBITING HSPA12B EXPRESSION VIA GPR81 DEPENDENT SIGNALING IN SEPSIS**

Min Fan^1,2†^, Kun Yang^1,2†^, Xiaohui Wang^1,2^, Xia Zhang^1^, Jingjing Xu^1^, Fei Tu^1^, P. Spencer Gill^1,2^, Tuanzhu Ha^1,2^, David L. Williams^1,2^, Chuanfu Li^1,2^*

^1^Department of Surgery, James H. Quillen College of Medicine, East Tennessee State University, Johnson City, TN 37614, USA

^2^Center of Excellence in Inflammation, Infectious Disease and Immunity, East Tennessee State University, Johnson City, TN 37614, USA

† Equal contribution to the study.

*Corresponding author:

Chuanfu Li, M.D

Department of Surgery, East Tennessee State University

Johnson City, TN 37614

Phone: 423-439-6349

Fax: 423-439-6259

Email: [Li@etsu.edu](mailto:Li@etsu.edu)

Sources of Funding: This work was supported, in part, by National Institutes of Health grants HL071837 (CL), HL153270 (CL), GM083016 (CL, DLW), GM119197 (DLW), C06RR0306551 (ETSU) and American Heart Association Predoctoral Fellowship 20PRE35120345 (MF). The authors have no conflicts of interest to declare.

Abbreviated Title: Lactate impairs vascular integrity via HSPA12B

**Abstract**

**Introduction**: Sepsis impaired vascular integrity results in multiple organ failure. Circulating lactate level is positively correlated with sepsis induced mortality. We investigated whether lactate plays a role in causing endothelial barrier dysfunction in sepsis.

**Methods**: Polymicrobial sepsis was induced in mice by cecal ligation and puncture (CLP). Lactic acid was injected intraperitoneally (pH 6.8, 0.5g/kg body weight) 6 hours after CLP or sham surgery. To elucidate the role of HSPA12B, wild type (WT), HSPA12B transgenic (HSPA12B-Tg) and endothelial HSPA12B deficient (eHSPA12B^-/-^) mice were subjected to CLP or sham surgery. To suppress lactate signaling, 3OBA (120 µM) was injected intraperitoneally 3 hours prior to surgery. Vascular permeability was evaluated with the Evans Blue Dye penetration assay.

**Results**: We found that administration of lactate elevated CLP induced vascular permeability. Vascular endothelial cadherin (VE-cadherin), Claudin5 and zonula occluden (ZO)-1 play a crucial role in the maintenance of endothelial cell junction and vascular integrity. Lactate administration significantly decreased VE-cadherin, Claudin5 and ZO-1 expression in the heart of septic mice. Our *in vitro* data showed that lactate (10 mM) treatment disrupted of VE-cadherin, Claudin5 and ZO-1 in endothelial cells. Mechanistically, we observed that lactate promoted VE-cadherin endocytosis by reducing the expression of HSPA12B. Overexpression of HSPA12B prevented lactate induced VE-cadherin disorganization. GPR81 is a specific receptor for lactate. Inhibition of GPR81 with its antagonist 3OBA attenuated vascular permeability and reversed HSPA12B expression in septic mice.

**Conclusions**: The present study demonstrated a novel role of lactate in promoting vascular permeability by decreasing VE-cadherin junctions and tight junctions in endothelial cells. The deleterious effects of lactate in vascular hyperpermeability are mediated via HSPA12B and GPR81-dependent signaling.

**Key words**: Lactate, vascular permeability, heat shock protein A12B, VE-cadherin, tight junction, polymicrobial sepsis

**Introduction**

Sepsis is defined as a dysregulated inflammatory response induced by an infection, which is associated with life-threating organ dysfunction ^1,2^. Endothelial hyperpermeability in response to sepsis and septic shock is a major contributor to multiple organ failure ^3,4^. Adherens junctions and tight junctions play an important role in regulating endothelial barrier integrity ^5^. Adhesion of vascular endothelial (VE)-cadherin, a key component of adherens junctions, is the major adhesion process in vascular development ^5,6^. Insufficient expression of the tight junction molecules, Claudin5 and zonula occluden (ZO)-1, disrupts endothelial cell-cell connection and increases endothelial permeability ^7,8^.

As a biomarker of septic shock, clinical studies reveal that serum lactate levels are closely associated with the morbidity and mortality of sepsis ^9^. Inhibition of aerobic glycolysis by either pyruvate kinase M2 (PKM2) ^10^ or 2-deoxy-Dglycose (2-DG) ^11^ decreases lactate production and improves survival rate in experimental model of sepsis induced by cecal ligation and puncture (CLP). In addition, our recent study has revealed that lactate promotes the production of inflammatory cytokine TNF-α and IL-6 ^12^, as well as release of exosomal high mobility group box1 (HMGB1) from macrophage, which can induce vascular endothelium permeability in experimental sepsis ^13^. However, whether lactate has a direct effect on endothelial permeability remains unclear.

Heat shock protein A12B (HSPA12B), a member of HSP70 family, is predominantly expressed in endothelium and required for angiogenesis ^14,15^. Our group has uncovered that deficiency of endothelial HSPA12B impairs angiogenesis in a mouse model of myocardial infarction ^16^ and induces intercellular adhesion molecule (ICAM)-1 and vascular cell adhesion molecule (VCAM)-1 expression in experimental sepsis ^17^. On the contrary, overexpression of HSPA12B promotes endothelial cell angiogenesis and attenuates adhesion molecular expression. Little is known about the role of HSPA12B in endothelial adherens junction and tight junction and its relationship with lactate in polymicrobial sepsis.

In the current study, we investigated whether lactate plays a role in endothelial permeability after sepsis and, if so, by what mechanism(s). We found that administration of lactate or depletion of endothelial cell HSPA12B exacerbates sepsis induced organ dysfunction and vascular hyperpermeability by impairing VE-cadherin junctions and tight junctions. Importantly, we observed that lactate reduced HSPA12B expression and HSPA12B is required for lactate regulated VE-cadherin junctions and tight junctions in endothelial cells. In addition, inhibition of lactate signaling reverses HSPA12B expression and improves vascular integrity after sepsis.

**Materials and Methods**

**Animals.** Endothelial cell specific HSPA12B deficient (eHSPA12B^-/-^) mice and HSPA12B transgenic (Tg) mice were constructed as described in our previous studies ^16,18^. Wild type (WT) C57BL/6 mice were purchased from Jackson Laboratory (Indianapolis, IN). All mice were maintained and bred at the Division of Laboratory Animal Resources at East Tennessee State University (ETSU). All experimental procedures were performed in accordance with the Guide for the Care and Use of Laboratory Animals published by the National Institutes of Health (NIH Publication, 8th Edition, 2011) and approved by the ETSU Committee on Animal Care (A3203-01).

**Polymicrobial sepsis model.** Male mice (8-10 weeks) were subjected to cecal ligation and puncture (CLP) to induce polymicrobial sepsis as previously described ^13^. Briefly, mice were anaesthetized and maintained by inhalation of 1.5-2% isoflurane driven by 100% oxygen flow. Following midline incision, the mouse cecum was ligated between 3rd and 4th vascular arcade with a 4-0 silk suture and punctured with a 25-gauge needle. Sham operation is performed by isolation of the cecum without ligation and puncture. A single dose of resuscitative fluid saline was administrated by subcutaneous injection after surgery. Mice were recovered in prewarmed cages (37°C).

***In vivo* treatments**. To investigate whether elevated serum lactate levels contribute to vascular barrier dysfunction, lactic acid (Millipore Sigma, pH 6.8, 0.5 g/kg body weight) was injected intraperitoneally (i.p.) 6 h after surgery. The dose of lactate is adjusted based on a previous publication ^19^ and was tested on our recent publications ^12,13^. In addition, this dose of lactate is non-lethal and does not cause acidosis ^20^. Serum lactate levels were measured by Lactate Assay Kit (Millipore Sigma, MAK064). In separate experiments, 3‐hydroxy‐butyrate (3OBA) (Millipore Sigma, 120 µM) were administrated to mice 3 h before CLP or sham surgical operation to block GPR81 signaling. The dose of 3-OBA was determined based on our pilot study and previous report ^13^.

**Echocardiography.** We performed echocardiography on mice 24 h after CLP to test cardiac function according to our previous studies ^18,21^. Left ventricular (LV) wall thickness, LV end-systolic diameter and LV end-diastolic diameter were examined by M-mode tracings. Thereafter, percent fractional shortening (FS%) and ejection fraction (EF%) were calculated.

**Organ injury measurement.** Serum creatinine (Millipore Sigma, MAK080) and aspartate aminotransferase (AST) (Millipore Sigma, MAK055) levels were measured by commercial kits according to the manufacturer’s protocols.

**Vascular permeability assay.** *In vivo* vascular permeability was assessed by the Evans Blue Dye (EBD) assay. 24 hours after CLP or sham surgery, 0.5% Evans blue (in PBS) was injected via penile vein 30 minutes before mice were sacrificed. Subsequently, mice were perfused with PBS via left ventricle to remove the intravascular dye. Livers and kidneys were removed and dried for overnight. EBD was extracted in formamide by incubation of tissues overnight at 55°C. EBD levels in tissue supernatant was measured by spectrophotometric analysis at 610 nm ^22,23^.

**Endothelial cell culture.** Human umbilical vein endothelial cells (HUVECs) (ATCC, PCS-100-013) were cultured in Vascular Cell Basal Medium (ATCC, PCS-100-030) supplemented with growth factors and 5% Fetal Bovine Serum (FBS) at 37°C with 5% CO2. HUVECs were treated with 10 mM L-lactic acid for 2 or 6 h.

**Cell transfection**. HUVECs were cultured to 70% confluence and were transfected with adenoviral HSPA12B (Ad-HSPA12B, MOI=10) or adenoviral GFP (Ad-GFP). 6 hours after transfection, cells were washed with PBS and incubated with fresh medium overnight before lactate treatment.

**Immunofluorescent (IF) staining.** Heart tissues were collected after euthanasia, fixed in 4% paraformaldehyde, embedded in tissue processing medium (O.C.T.) and cut horizontally at a 10 mm thickness. The slides were stained with specific anti-VE-Cadherin antibody (1:200 dilution, Santa Cruz Biotechnology, sc-9989) at 4°C for overnight. HUVECs obtained from *in vitro* cell culture were fixed with 3.7% formaldehyde for 20 min, permeabilized with 0.1% Triton® X-100 for 20 min, followed by blocking with 3%BSA for 30 min at room temperature. Subsequently, cells were incubated with anti-VE-Cadherin antibody (1:200 dilution, Santa Cruz Biotechnology, sc-9989) at 4°C for overnight. Alexa Fluor 594 secondary antibody (1:500 dilution in PBS, Invitrogen, A-11005) was added to the section to visualize the staining. DAPI (blue) was used to counterstain the nuclei. The stained-sections and cells were measured using Confocal Microscope (Leica).

**ELISA.** The serum levels of pro-inflammatory cytokines (TNFα and IL6) following sepsis were measured using commercially available ELISA kits (PeproTech, US) according to the instruction provided by the manufacturer.

**Western blot.** Western blot was performed as described previously (7). Briefly, cellular proteins were prepared in ice-cold RIPA buffer containing protease inhibitor and quantified with Pierce BCA protein assay kit (ThermoFisher, 23225). Proteins were separated on 10% SDS-PAGE and transferred onto nitrocellulose blotting membranes (GE Healthcare, 10600003). The membranes were incubated with appropriate primary antibodies (1:1000 dilution in 5% BSA) followed by the peroxidase-conjugated secondary antibody (1:5000 dilution in 5% non-fat milk). The signals were quantified using G: Box gel imaging system by Syngene. The following primary antibodies were used in the present study: anti-VE-Cadherin antibody (1:200 dilution, Santa Cruz Biotechnology, sc-9989), anti-HSPA12B antibody (1:1000 dilution, a gift from Dr. Zhihua Han (ETSU, Johnson city)), anti-Claudin5 antibody (1:1000 dilution, Abcam, ab131259), anti-ZO-1 antibody (1:1000 dilution, Cell Signaling Technology, 13663s), anti-β-actin antibody (1:1000 dilution, Cell Signaling Technology, 3700s), anti-GAPDH antibody (1:1000 dilution, Cell Signaling Technology, 2118s).

**Quantitative Real-time PCR (qRT-PCR).** Total mRNA was isolated from cultured cells using RNAzol®RT (Molecular Research Center, RN 190) in accordance with the manufacture’s protocol as described previously ^24^. The mRNA was converted to cDNA by High Capacity cDNA Transcription kit (Applied Biosystems, 4368814). The mRNA levels of *VE-Cadherin, Claudin5* and *ZO1* were measured using SYBR green mix (Sigma-Aldrich, KCQS00) system and quantified with the 2 (-ΔΔct) relative quantification method that were normalized to *β-Actin*.

**Statistics.** Data are expressed as means ± SD. Comparisons of data between groups were made using 2 tailed t-test or one-way analysis of variance (ANOVA) followed by Tukey’s procedure for multiple range tests. P < 0.05 was considered to be significant.

**Results**

**Lactate induces organ dysfunction and vascular permeability in sepsis.**

Accumulating evidence has revealed that increased lactate levels correlate with multiple organ dysfunction in septic patients ^9,25,26^. To investigate whether lactate is involved in the development of organ dysfunction during sepsis, we administrated lactate (0.5g/kg body weight) via i.p. injection 6 hours after CLP or sham surgery and tissues were collected for subsequent analysis (**Figure 1A**). We observed that CLP strongly induced neutrophil infiltration in the spleen (**Figure S1A, http://links.lww.com/SHK/B501**) and increased serum levels of TNFα and IL6 (**Figure S1B and C, http://links.lww.com/SHK/B501**) as compared to sham mice. In consistent with previous reports, CLP increased serum lactate levels (**Figure 1B**), and induced liver and kidney injury as evidenced by increased serum AST (6.58±1.17 vs 9.47±1.51) and creatinine (17.5±5.43 vs 44.0±2.95) levels when compared with sham group (**Figure 1C** and **D**) ^11,27^. Importantly, lactate supplementation further increased serum AST and creatinine levels by 41.4% and 64.3%, respectively, when compared with CLP mice with PBS supplementation (**Figure 1C** and **D**). In addition, lactate administration suppressed pro-inflammatory cytokine (TNFα and IL6) production following sepsis (**Figure S1B and C, http://links.lww.com/SHK/B501**), which is consistent with our previous findings ^12^. These data suggest that lactate promotes organ dysfunction during sepsis. Vascular leakage leads to tissue edema and impaired vascular perfusion, which consequently contributes to organ dysfunction in critical illness, such as sepsis ^3,28^.Next, we assessed whether lactate affected vascular permeability after CLP sepsis by Evans blue assay ^22^. As shown in **Figure 1E** and **F**, CLP increased vascular permeability in liver and kidney as evidenced by increased Evans blue dye (EBD) penetration to the tissues. Supplementation of lactate further increased liver and kidney vascular permeability in sepsis mice (**Figure 1E** and **1F**). Notably, lactate supplementation did not significantly increased serum lactate levels in sham operated mice (**Figure 1B**), which is in agreement with a recent study showing that lactate levels were back to baseline concentrations 30 minutes after lactate i.p. injection ^19^. Clearance of lactate from the circulation is primarily mediated by the liver (60%) and kidney (30%) ^29^. Lactate is metabolized and cleared rapidly in sham operated mice with liver and kidney function (**Figure 1C** and **1D**). Therefore, lactate supplementation did not cause vascular permeability in sham mice (**Figure 1E** and **1F**). Together, our data suggests that lactate induces vascular permeability and contributes to organ dysfunction in sepsis.

**Lactate impairs VE-cadherin junctions and tight junctions in endothelial cells.**

As a major adherens junctional protein, vascular endothelial (VE)-cadherin plays a crucial role in maintaining endothelial cell connection and stability ^30,31^. We next determined whether lactate can alter VE-cadherin integrity. As shown in **Figure 2A**, there was a reduction of VE-cadherin expression in septic hearts, when compared with the sham control group. Moreover, lactate treatment further disrupted the expression of VE-cadherin after sepsis (**Figure 2A**). Besides adherens junctions, tight junctions are also important in maintaining organ integrity ^32^. We examined the expression of the tight junction protein Claudin5 in the heart and detected lower Claudin5 expression with lactate treatment than septic control mice (**Figure 2A**), indicating that lactate exerts an additive effect by impairing tight junctions in the myocardium after sepsis.

To further confirm the role of lactate in endothelial cell function, we treated HUVECs with lactate *in vitro* and investigated the expression of VE-cadherin, Claudin5 and ZO-1. In accordance with our *in vivo* finding, lactate administration decreased VE-cadherin, Claudin5 and ZO-1 levels in endothelial cells (**Figure 2B**).

**Lactate suppresses HSPA12B expression in endothelial cells.**

Heat shock protein A12B (HSPA12B) is a recently discovered protein that is primarily expressed in endothelial cells ^14,15^. We and others have previously showed that HSPA12B is required for endothelial cell angiogenesis and adhesion ^15,16,33^. We found that the expression of HSPA12B in the heart decreased remarkably after CLP sepsis (**Figure 3A**). Interestingly, treatment with supplemental lactate further downregulated HSPA12B expression following sepsis (**Figure 3A**). In addition, administration of lactate disrupted HSPA12B expression in endothelial cells (**Figure 3B**). Taken together, our findings suggest that HSPA12B may play a critical role in lactate impaired endothelial barrier leakage.

**Endothelial cell specific deletion of HSPA12B exacerbates organ dysfunction and promotes vascular hyperpermeability after sepsis.**

Next, we induced CLP sepsis in wild type (WT), HSPA12B transgenic and endothelial cell specific HSPA12B deficient (eHSPA12B^-/-^) mice and tested cardiac function 24 hours after surgery. CLP reduced the percent fractional shortening (FS%) and ejection fraction (EF%) (**Figure 4A-D**). However, overexpression of HSPA12B reversed FS% and EF% levels after CLP (**Figure 4A** and **B**). In contrast, depletion of endothelial cell HSPA12B further suppressed FS% and EF% levels (**Figure 4C** and **D**). These data indicate that HSPA12B is required for maintainence of cardiac function after sepsis. Moreover, serum AST and creatinine levels were higher in eHSPA12B^-/-^ CLP mice than in WT CLP mice (**Figure 4E** and **F**), suggesting that inhibition of HSPA12B worsens liver and kidney injury after sepsis.

We then addressed whether HSPA12B participates in vascular permeability following sepsis. **Figure 4G-I** showed that HSPA12B deficiency enhanced sepsis induced endothelial hyperpermeability. Additionally, the levels of Claudin5 and ZO-1 were significantly lower in eHSPA12B^-/-^ hearts than in the heats of WT septic mice (**Figure 5A**). **Figure 5B** and **C** showed that the positive staining of VE-cadherin was decreased in both hearts and lung of eHSPA12B^-/-^ mice after CLP, when compared with WT CLP mice. These data reveal that HSPA12B is required for maintaining tight junctions and VE-cadherin junctions after sepsis.

**HSPA12B is required for lactate decreased VE-cadherin junctions and tight junctions in endothelial cells.**

To elucidate whether HSPA12B plays a role in lactate induced endothelial permeability, we transfected endothelial cells with adenoviral HSPA12B (Ad-HSPA12B) or Ad-GFP before lactate administration. As shown in **Figure 6A**, overexpression of HSPA12B attenuated the down regulation of VE-cadherin, Claudin5 and ZO-1 expression induced by lactate. Similarly, transfection of Ad-HSPA12B upregulated mRNA levels of VE-cadherin, Claudin5 and ZO-1 that were repressed by lactate (**Figure 6B-D**). When compared with Ad-GFP transfection, the integrity of VE-cadherin was reversed by Ad-HSPA12B transfection after lactate treatment (**Figure 6E**). These results showed that HSPA12B is required for lactate regulated VE-cadherin junctions and tight junctions.

**Inhibition of GPR81/lactate signaling induces HSPA12B expression decreased by sepsis and improves vascular integrity.**

Lactate is regarded as a signaling molecule that interacts with G protein-coupled receptor 81 (GPR81) ^34^. To determine the role of GPR81/lactate signaling, mice were i.p. injected of a GPR81 inhibitor, 3‐hydroxy‐butyrate (3OBA) ^35^, before CLP or sham surgery. Administration of 3OBA enhanced HSPA12B expression in the presence of sepsis (**Figure 7A**). In addition, 3OBA treatment attenuated sepsis induced liver and kidney EBD penetration (**Figure 7B** and **C**), suggesting that inhibition of GPR81/lactate signaling improves vascular permeability after sepsis. Moreover, 3-OBA administration also improved survival outcomes of septic mice (**Figure 7D**).

**Discussion**

In this study, we describe a role for lactate as a mediator of vascular permeability and multiple organ dysfunction in sepsis. Our findings show that lactate disrupts VE-cadherin integrity and decreases the expressions of VE-cadherin and tight junction proteins (Claudin 5 and ZO-1) in endothelial cells. We also observed that administration of supplemental lactate exacerbated sepsis-induced decreases in HSPA12B expression. Endothelial specific deletion of HSPA12B exhibited vascular hyperpermeability via downregulation of VE-cadherin and tight junction protein levels, and worsened multiple organ dysfunction during polymicrobial sepsis. In contrast, adenovirus-mediated overexpression of HSPA12B attenuated the effect of lactate on VE-cadherin, Claudin5 and ZO1 in endothelial cells. Importantly, pharmacological inhibition of lactate/GPR81 signaling attenuated sepsis-induced HSPA12B downregulation, vascular permeability and improved survival outcome of septic mice. This suggests that it may be possible to prevent and/or treat lactate induced organ injury and mortality in sepsis.

Sepsis is a complex syndrome initiated by infection and characterized by multiple organ dysfunction. Vascular endothelial damage, resulting from the invading microbes and systemic pro-inflammation, contributes to the pathogenesis of multiple organ dysfunction during sepsis ^36-38^. Therefore, understanding the mechanisms that lead to vascular injury would be an important advance in sepsis management.

We and others have demonstrated that glycolysis-derived lactate is a critical effector metabolite that triggers various cell signaling pathways in regulating innate immune responses during experimental sepsis ^12,13,20,39,40^. Of note, a recent study by Khatib-Massalha *et al.* shows that elevated levels of bone marrow (BM) lactate increase BM vascular permeability, leading to enhanced neutrophil mobilization during infection ^20^. To examine whether systemic elevation of lactate levels could induce vascular permeability, mice were subjected to CLP-induced sepsis followed by intraperitoneal administration of lactate. We observed that CLP induced vascular permeability in the liver and kidney tissues. Lactate supplementation further promoted vascular permeability in CLP sepsis mice. Consistent with these observations, our data show that lactate supplementation worsened CLP-induced liver and kidney dysfunction.

Previous studies have reported that HSPA12B is required to maintain endothelium homeostasis in the mouse model of sepsis and sepsis-induced cardiovascular diseases ^17,18,41^. In addition, it is reported that the circulating HSPA12B levels are higher in patients with severe sepsis (patients with at least one organ dysfunction within 24 hours after inclusion, N = 66) than in patients with sepsis (N = 21), suggesting that HSPA12B could be a biomarker of endothelial cell injury in sepsis ^42^. In the present study, we found that HSAP12B is an essential mediator in lactate-induced downregulation of VE-cadherin, Claudin5 and ZO-1 in endothelial cells. Of greater significance, genetic depletion of HSPA12B in endothelial cells renders mice more susceptible to sepsis-induced downregulation of adhesion molecules and tight junction proteins, vascular hypermutability and multiple organ dysfunction. This observation is consistent with our recent studies showing that endothelial cell-specific deletion of HSPA12B increased mortality rate of septic mice ^17,41^. Conversely, adenovirus-mediated overexpression of HSPA12B preserved the expressions of VE-cadherin, Claudin5 and ZO-1 in lactate-treated endothelial cells. In addition, pharmacologically inhibiting the lactate receptor, GPR81, attenuated sepsis-induced decreases in HSPA12B expression, vascular permeability and improved survival outcome of septic mice.

Several limitations are presented in our present study. Although we show that overexpression of HSPA12B preserved the expression of VE-cadherin, Claudin5 and ZO-1 in lactate-treated endothelial cells, the underlying mechanisms are unclear. Our previous study revealed that HSPA12B cooperates with transcription regulator YAP in regulating the transcription of genes associated with angiogenesis ^16^. It is reported that YAP regulates adhesion junction dynamics in endothelial cells and is required for the formation of barrier integrity of endothelium ^43,44^. Therefore, it is intriguing to speculate the involvement of YAP signaling in lactate-induced vascular permeability in future studies. In addition, accumulating evidence indicate that lactate regulates the response of immune cells to infection ^12,13,45^. Therefore, it is imperative to investigate whether lactate could alter immune cell functions, such as bacterial clearance, thereby consequently contributing to vascular permeability.

In conclusion, the findings of this study delineate the essential role of lactate in promoting vascular permeability by downregulating HSPA12B in endothelial cells during sepsis. Suppression of lactate/GPR81 signaling is effective in reducing vascular permeability and improving organ function in polymicrobial sepsis. Therefore, our study not only provides molecular basis for the lactate as critical biomarker in sepsis prognosis but also suggests that blocking lactate/GPR81 signaling by GPR81 inhibitors may prevent lethality in sepsis.

**References**

1. Singer M, Deutschman CS, Seymour CW, Shankar-Hari M, Annane D, Bauer M, Bellomo R, Bernard GR, Chiche JD, Coopersmith CM, et al. The Third International Consensus Definitions for Sepsis and Septic Shock (Sepsis-3). *Jama-J Am Med Assoc.* 315(8):801-810, 2016.

2. Cecconi M, Evans L, Levy M, Rhodes A. Sepsis and septic shock. *Lancet.* 392(10141):75-87, 2018.

3. Ince C, Mayeux PR, Nguyen T, Gomez H, Kellum JA, Ospina-Tascon GA, Hernandez G, Murray P, De Backer D, Workgroup AX. The Endothelium in Sepsis. *Shock.* 45(3):259-270, 2016.

4. Li Z, Yin M, Zhang H, Ni W, Pierce RW, Zhou HJ, Min W. BMX Represses Thrombin-PAR1-Mediated Endothelial Permeability and Vascular Leakage During Early Sepsis. *Circ Res.* 126(4):471-485, 2020.

5. Komarova YA, Kruse K, Mehta D, Malik AB. Protein Interactions at Endothelial Junctions and Signaling Mechanisms Regulating Endothelial Permeability. *Circ Res.* 120(1):179-206, 2017.

6. Breier G, Breviario F, Caveda L, Berthier R, Schnurch H, Gotsch U, Vestweber D, Risau W, Dejana E. Molecular cloning and expression of murine vascular endothelial-cadherin in early stage development of cardiovascular system. *Blood.* 87(2):630-641, 1996.

7. Li B, Yu Y, Liu K, Zhang Y, Geng Q, Zhang F, Li Y, Qi J. beta-Hydroxybutyrate inhibits histone deacetylase 3 to promote claudin-5 generation and attenuate cardiac microvascular hyperpermeability in diabetes. *Diabetologia.* 64(1):226-239, 2021.

8. Tornavaca O, Chia M, Dufton N, Almagro LO, Conway DE, Randi AM, Schwartz MA, Matter K, Balda MS. ZO-1 controls endothelial adherens junctions, cell-cell tension, angiogenesis, and barrier formation. *J Cell Biol.* 208(6):821-838, 2015.

9. Nolt B, Tu F, Wang X, Ha T, Winter R, Williams DL, Li C. Lactate and Immunosuppression in Sepsis. *Shock.* 49(2):120-125, 2018.

10. Xie M, Yu Y, Kang R, Zhu S, Yang L, Zeng L, Sun X, Yang M, Billiar TR, Wang H, et al. PKM2-dependent glycolysis promotes NLRP3 and AIM2 inflammasome activation. *Nat Commun.* 7:13280, 2016.

11. Zheng Z, Ma H, Zhang X, Tu F, Wang X, Ha T, Fan M, Liu L, Xu J, Yu K, et al. Enhanced Glycolytic Metabolism Contributes to Cardiac Dysfunction in Polymicrobial Sepsis. *J Infect Dis.* 215(9):1396-1406, 2017.

12. Yang K, Xu J, Fan M, Tu F, Wang X, Ha T, Williams DL, Li C. Lactate Suppresses Macrophage Pro-Inflammatory Response to LPS Stimulation by Inhibition of YAP and NF-kappaB Activation via GPR81-Mediated Signaling. *Front Immunol.* 11:587913, 2020.

13. Yang K, Fan M, Wang X, Xu J, Wang Y, Tu F, Gill PS, Ha T, Liu L, Williams DL, et al. Lactate promotes macrophage HMGB1 lactylation, acetylation, and exosomal release in polymicrobial sepsis. *Cell Death Differ.* 2021.

14. Han Z, Truong QA, Park S, Breslow JL. Two Hsp70 family members expressed in atherosclerotic lesions. *Proc Natl Acad Sci U S A.* 100(3):1256-1261, 2003.

15. Steagall RJ, Rusinol AE, Truong QA, Han Z. HSPA12B is predominantly expressed in endothelial cells and required for angiogenesis. *Arterioscler Thromb Vasc Biol.* 26(9):2012-2018, 2006.

16. Fan M, Yang K, Wang X, Wang Y, Tu F, Ha T, Liu L, Williams DL, Li C. Endothelial cell HSPA12B and yes-associated protein cooperatively regulate angiogenesis following myocardial infarction. *JCI insight.* 2020.

17. Zhang X, Wang X, Fan M, Tu F, Yang K, Ha T, Liu L, Kalbfleisch J, Williams D, Li C. Endothelial HSPA12B Exerts Protection Against Sepsis-Induced Severe Cardiomyopathy via Suppression of Adhesion Molecule Expression by miR-126. *Frontiers in Immunology.* 11:566, 2020.

18. Zhou H, Qian J, Li C, Li J, Zhang X, Ding Z, Gao X, Han Z, Cheng Y, Liu L. Attenuation of cardiac dysfunction by HSPA12B in endotoxin-induced sepsis in mice through a PI3K-dependent mechanism. *Cardiovasc Res.* 89(1):109-118, 2011.

19. Haugen OP, Vallenari EM, Belhaj I, Smastuen MC, Storm-Mathisen J, Bergersen LH, Amellem I. Blood lactate dynamics in awake and anaesthetized mice after intraperitoneal and subcutaneous injections of lactate-sex matters. *PeerJ.* 8:e8328, 2020.

20. Khatib-Massalha E, Bhattacharya S, Massalha H, Biram A, Golan K, Kollet O, Kumari A, Avemaria F, Petrovich-Kopitman E, Gur-Cohen S, et al. Lactate released by inflammatory bone marrow neutrophils induces their mobilization via endothelial GPR81 signaling. *Nat Commun.* 11(1):3547, 2020.

21. Lu C, Ren D, Wang X, Ha T, Liu L, Lee EJ, Hu J, Kalbfleisch J, Gao X, Kao R, et al. Toll-like receptor 3 plays a role in myocardial infarction and ischemia/reperfusion injury. *Biochim Biophys Acta.* 1842(1):22-31, 2014.

22. Radu M, Chernoff J. An in vivo assay to test blood vessel permeability. *J Vis Exp.* (73):e50062, 2013.

23. Wick MJ, Harral JW, Loomis ZL, Dempsey EC. An Optimized Evans Blue Protocol to Assess Vascular Leak in the Mouse. *J Vis Exp.* (139), 2018.

24. Wang X, Ha T, Liu L, Hu Y, Kao R, Kalbfleisch J, Williams D, Li C. TLR3 Mediates Repair and Regeneration of Damaged Neonatal Heart through Glycolysis Dependent YAP1 Regulated miR-152 Expression. *Cell Death Differ.* 25(5):966-982, 2018.

25. Bakker J, Gris P, Coffernils M, Kahn RJ, Vincent JL. Serial blood lactate levels can predict the development of multiple organ failure following septic shock. *Am J Surg.* 171(2):221-226, 1996.

26. Jansen TC, van Bommel J, Woodward R, Mulder PG, Bakker J. Association between blood lactate levels, Sequential Organ Failure Assessment subscores, and 28-day mortality during early and late intensive care unit stay: a retrospective observational study. *Crit Care Med.* 37(8):2369-2374, 2009.

27. Yang L, Xie M, Yang M, Yu Y, Zhu S, Hou W, Kang R, Lotze MT, Billiar TR, Wang H, et al. PKM2 regulates the Warburg effect and promotes HMGB1 release in sepsis. *Nat Commun.* 5:4436, 2014.

28. Dupont A, Rauch A, Staessens S, Moussa M, Rosa M, Corseaux D, Jeanpierre E, Goutay J, Caplan M, Varlet P, et al. Vascular Endothelial Damage in the Pathogenesis of Organ Injury in Severe COVID-19. *Arterioscler Thromb Vasc Biol.* 41(5):1760-1773, 2021.

29. Levy B. Lactate and shock state: the metabolic view. *Curr Opin Crit Care.* 12(4):315-321, 2006.

30. Lampugnani MG, Corada M, Caveda L, Breviario F, Ayalon O, Geiger B, Dejana E. The molecular organization of endothelial cell to cell junctions: differential association of plakoglobin, beta-catenin, and alpha-catenin with vascular endothelial cadherin (VE-cadherin). *J Cell Biol.* 129(1):203-217, 1995.

31. Tamura K, Shan WS, Hendrickson WA, Colman DR, Shapiro L. Structure-function analysis of cell adhesion by neural (N-) cadherin. *Neuron.* 20(6):1153-1163, 1998.

32. Balda MS, Matter K. Tight junctions as regulators of tissue remodelling. *Curr Opin Cell Biol.* 42:94-101, 2016.

33. Hu G, Tang J, Zhang B, Lin Y, Hanai J, Galloway J, Bedell V, Bahary N, Han Z, Ramchandran R, et al. A novel endothelial-specific heat shock protein HspA12B is required in both zebrafish development and endothelial functions in vitro. *J Cell Sci.* 119(Pt 19):4117-4126, 2006.

34. Roland CL, Arumugam T, Deng D, Liu SH, Philip B, Gomez S, Burns WR, Ramachandran V, Wang H, Cruz-Monserrate Z, et al. Cell surface lactate receptor GPR81 is crucial for cancer cell survival. *Cancer Res.* 74(18):5301-5310, 2014.

35. Shen Z, Jiang L, Yuan Y, Deng T, Zheng YR, Zhao YY, Li WL, Wu JY, Gao JQ, Hu WW, et al. Inhibition of G Protein-Coupled Receptor 81 (GPR81) Protects Against Ischemic Brain Injury. *Cns Neurosci Ther.* 21(3):271-279, 2015.

36. Chang JC. Sepsis and septic shock: endothelial molecular pathogenesis associated with vascular microthrombotic disease. *Thromb J.* 17:10, 2019.

37. Peters K, Unger RE, Brunner J, Kirkpatrick CJ. Molecular basis of endothelial dysfunction in sepsis. *Cardiovasc Res.* 60(1):49-57, 2003.

38. London NR, Zhu W, Bozza FA, Smith MC, Greif DM, Sorensen LK, Chen L, Kaminoh Y, Chan AC, Passi SF, et al. Targeting Robo4-dependent Slit signaling to survive the cytokine storm in sepsis and influenza. *Sci Transl Med.* 2(23):23ra19, 2010.

39. Yang L, Xie M, Yang M, Yu Y, Zhu S, Hou W, Kang R, Lotze MT, Billiar TR, Wang H, et al. PKM2 regulates the Warburg effect and promotes HMGB1 release in sepsis. *Nature Communications.* 5, 2014.

40. Errea A, Cayet D, Marchetti P, Tang C, Kluza J, Offermanns S, Sirard JC, Rumbo M. Lactate Inhibits the Pro-Inflammatory Response and Metabolic Reprogramming in Murine Macrophages in a GPR81-Independent Manner. *PLoS One.* 11(11):e0163694, 2016.

41. Tu F, Wang X, Zhang X, Ha T, Wang Y, Fan M, Yang K, Gill PS, Ozment TR, Dai Y, et al. Novel Role of Endothelial Derived Exosomal HSPA12B in Regulating Macrophage Inflammatory Responses in Polymicrobial Sepsis. *Frontiers in Immunology.* 11:825, 2020.

42. Zhang R, Wan XJ, Zhang X, Kang QX, Bian JJ, Yu GF, Wang JF, Zhu KM. Plasma HSPA12B is a potential predictor for poor outcome in severe sepsis. *PLoS One.* 9(6):e101215, 2014.

43. Kim J, Kim YH, Kim J, Park DY, Bae H, Lee DH, Kim KH, Hong SP, Jang SP, Kubota Y, et al. YAP/TAZ regulates sprouting angiogenesis and vascular barrier maturation. *J Clin Invest.* 127(9):3441-3461, 2017.

44. Neto F, Klaus-Bergmann A, Ong YT, Alt S, Vion AC, Szymborska A, Carvalho JR, Hollfinger I, Bartels-Klein E, Franco CA, et al. YAP and TAZ regulate adherens junction dynamics and endothelial cell distribution during vascular development. *Elife.* 7, 2018.

45. Ratter JM, Rooijackers HMM, Hooiveld GJ, Hijmans AGM, de Galan BE, Tack CJ, Stienstra R. In vitro and in vivo Effects of Lactate on Metabolism and Cytokine Production of Human Primary PBMCs and Monocytes. *Front Immunol.* 9:2564, 2018.

**Figure 1. Lactate administration induced organ dysfunction and vascular permeability after CLP sepsis.** Mice were subjected to CLP or sham surgery followed by PBS or Lactic acid (pH 6.8, 0.5 g/kg body weight) intraperitoneally (i.p.) injection. Twenty-four hours later, blood was collected (**A**) and serum was isolated to examine serum lactate (**B**), aspartate aminotransferase (AST) (**C**) and creatinine (**D**) levels. **E-F**, In separate experiments, 0.5% Evans blue was injected via penile vein 30 minutes before mice were sacrificed. Vascular permeability of liver and kidney were measured. N=3-5/group. ^*^ P< 0.05, ^**^ P <0.01, ^***^ P <0.001 compared with indicated group.

**Figure 2.** **Lactate administration decreased VE-cadherin junctions and tight junctions after CLP. A**, Mice were subjected to CLP or sham surgery followed by PBS or Lactic acid (pH 6.8, 0.5 g/kg body weight) i.p. injection. Twenty-four hours later, heart tissues were collected and protein was isolated. The levels of VE-cadherin and Claudin5 in the myocardium were examined by Western blot. N=4/group. **B**, HUVECs were treated with L-lactic acid (10mM) for 6 hours and protein of HUVECs were then isolated. The expression of VE-cadherin, Claudin5 and ZO-1 was examined by Western blot. N=3/group. ^*^ P< 0.05, ^**^ P <0.01, ^***^ P <0.001 compared with indicated group.

**Figure 3. Administration of lactate eliminated HSPA12B expression following sepsis.** **A**, Mice were subjected to CLP or sham surgery followed by PBS or Lactic acid (pH 6.8, 0.5 g/kg body weight) i.p. injection. Twenty-four hours later, heart tissues were collected and protein was isolated. The levels of HSPA12B in the myocardium were examined by Western blot. N=4/group. **B**, HUVECs were treated with L-lactic acid (10mM) for 6 hours and protein of HUVECs were then isolated. The expression of HSPA12B was examined by Western blot. N=3/group. ^*^ P< 0.05, ^***^ P <0.001 compared with indicated group.

**Figure 4. Endothelial cell specific depletion of HSPA12B elevated CLP induced organ dysfunction and endothelial hyperpermeability.** Wild type (WT), HSPA12B transgenic (Tg) and endothelial HSPA12B knockout (eHSPA12B^-/-^) mice were subjected to sham or CLP surgical operation. **A-D**, Cardiac function was examined by echocardiography 24 hours after surgery. **E-F**, Serum AST and creatinine levels were examined 24 hours after surgery. **G-I**, In separate experiments, 0.5% Evans blue was injected via penile vein 30 minutes before mice were sacrificed. Vascular permeability of liver, kidney and lung were measured. N=4-5/group. ^*^ P< 0.05, ^**^ P <0.01, ^***^ P <0.001 compared with indicated group.

**Figure 5. HSPA12B is required for maintaining VE-cadherin junctions and tight junctions after sepsis.** WT and eHSPA12B^-/-^ mice were subjected to sham or CLP surgery. Twenty-four hours later, heart and lung tissues were collected. **A**, The levels of Claudin5 and ZO-1 were examined by Western blot. Immunofluorescent staining was performed to investigate the integrity of VE-cadherin in the heart (**B**) and lung (**C**). N=4-5/group. ^*^ P< 0.05 compared with indicated group.

**Figure 6. HSPA12B is required for lactate decreased** **VE-cadherin junctions and tight junctions in endothelial cells.** Endothelial cells were transfected with Ad-HSPA12B or Ad-GFP followed by lactate administration (10mM). The expression of VE-cadherin, Claudin5 and ZO-1 was examined by Western blot (**A**) and qRT-PCR (**B-D**). **E**, The integrity of VE-cadherin in endothelial cells was measured by immunofluorescent staining. N=3-4/group. ^*^ P< 0.05, ^**^ P <0.01, ^***^ P <0.001 compared with indicated group.

**Figure 7. Administration of 3OBA protected against CLP blunted HSPA12B expression, induced hyperpermeability and mouse death rate.** Mice were treated with PBS or 3‐hydroxy‐butyrate (3OBA) (120 µM) by i.p. injection 3 hours before subjecting to CLP or sham surgery followed. **A**, Twenty-four hours later, heart tissues were collected and protein was isolated. The levels of HSPA12B in the myocardium were examined by Western blot. N=5/group. **B-C**, 0.5% Evans blue was injected via penile vein 30 minutes before mice were sacrificed. Vascular permeability of liver and kidney were measured. N=3-4/group. **D**, In separate experiments, mouse survival rate was measured (N=22/group). ^*^ P< 0.05, ^**^ P <0.01, ^***^ P <0.001 compared with indicated group.

**Supplementary Figure 1. Lactate suppresses pro-inflammatory cytokine production in sepsis. A**, Representative image of immunofluorescence staining of neutrophil (green color) in frozen sections of spleen tissue 24 hours following CLP. **B-C,** Lactate decreases serum levels of TNFα and IL6 in septic mice. N=6/group ^*^ P< 0.05, ^**^ P <0.01, ^***^ P <0.001 compared with indicated group.
