# Supplementary figures and images for: LACTATE IMPAIRS VASCULAR PERMEABILITY BY INHIBITING HSPA12B EXPRESSION VIA GPR81-DEPENDENT SIGNALING IN SEPSIS
Source: Shock. 2022 Sep 30;58(4):304–12. doi: 10.1097/SHK.0000000000001983 (PMC9584042; doi:10.1097/SHK.0000000000001983)

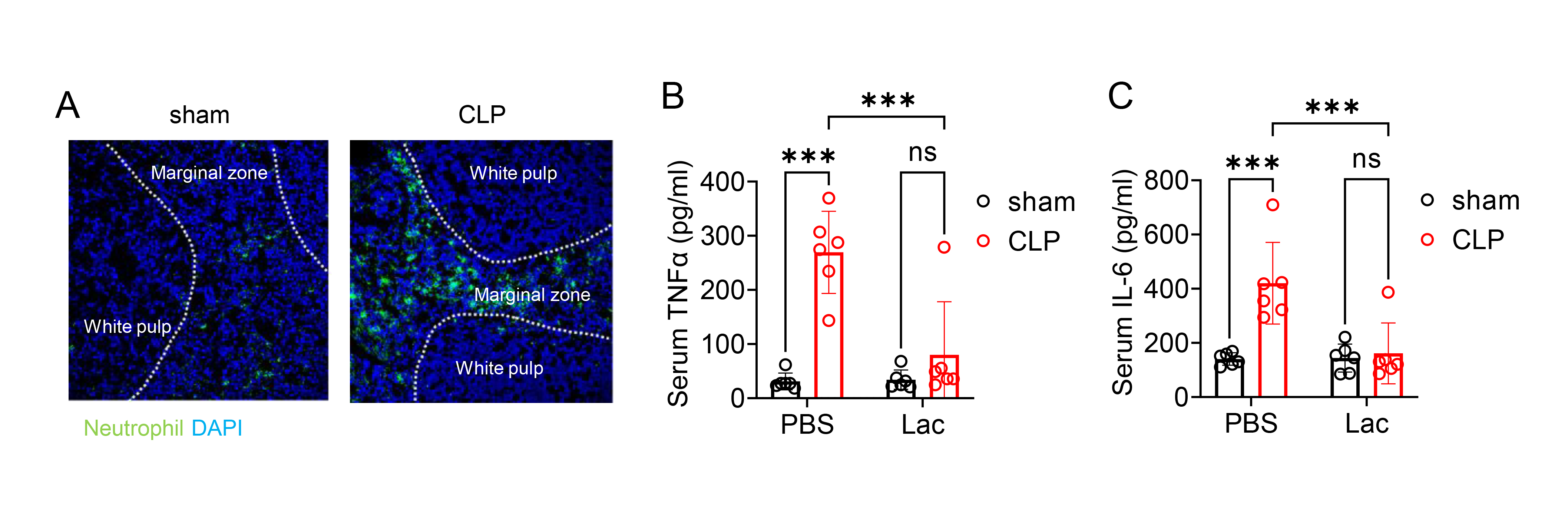

Supplement: SUPPLEMENTARY MATERIAL [file shock-58-304-s003.tif]
